# S-Adenosylmethionine Depletion as Potential Rate-Limiting Nexus of Redox–Epigenetic Crosstalk in Neural Tube Defects: A Mechanistic Theory and Experimental Hypothesis

**DOI:** 10.3390/cells15201896

**Published:** 2026-10-17

**Authors:** Boniface Ezenwata Echefu, Maria Becker, Asher Ornoy

**Affiliations:** 1 Department of Morphological Sciences and Teratology, Adelson School of Medicine, Ariel University, Ariel 40700, Israel; bonifacee@ariel.ac.il (B.E.E.); mariabe@ariel.ac.il (M.B.); 2 Hadassah Medical School, Hebrew University of Jerusalem, Jerusalem 91010, Israel

**Keywords:** cellular and molecular mechanisms, epigenetics, redox stress, neural tube defects, diabetes, valproic acid, S-adenosylmethionine, antioxidants

## Abstract

**Highlights:**

**What are the main findings?**

- Oxidative and nitrosative stress converge on SAMe depletion as a possible rate-limiting metabolic bottleneck that constrains methyltransferase activity and disrupts DNA and histone methylation programs essential for neural tube closure.
- Diverse environmental insults produce overlapping neurulation defects through a shared redox–one-carbon–epigenetic axis. SAMe deficiency and rescue in mice models provide preliminary experimental support for this proposed mechanism.

**What are the implications of the main findings?**

- SAMe insufficiency may function not merely as a methyl-donor deficit but as a central metabolic integrator of redox buffering and epigenetic fidelity, potentially positioning SAMe-centered metabolic control as a plausible target for NTD prevention that remains to be established in human pregnancy.
- Integrated quantification of SAMe, SAH, homocysteine, and GSH alongside locus-specific methylation profiling could help validate this model and determine whether these biomarkers identify pregnancies at increased risk before morphogenetic failure becomes irreversible.

**Abstract:**

S-adenosylmethionine (SAMe) depletion may represent a rate-limiting nexus linking oxidative/nitrosative stress to epigenetic failure during neurulation. In this review, we synthesize evidence that diverse teratogenic and metabolic insults, including valproic acid (VPA) exposure, maternal diabetes, and folate-dependent one-carbon imbalance, converge on a shared redox–metabolic–epigenetic axis that lowers SAMe availability, elevates S-adenosylhomocysteine, weakens glutathione buffering, and disrupts DNA and histone methylation/demethylation programs of genes required for neural tube closure. This framework explains how redox stress can impair key developmental pathways such as Wnt/β-catenin, sonic hedgehog, and Pax3 while promoting neuroepithelial apoptosis, abnormal proliferation, and failure of morphogenetic coordination. By integrating redox stress with chromatin regulation and one-carbon metabolism, the proposed model provides a unifying mechanism for neural tube defect pathogenesis and positions SAMe-centered metabolic control as a plausible therapeutic target. Definitive testing will require demonstrating that redox stress lowers embryonic SAMe before closure failure, that this precedes epigenetic dysregulation at neurulation genes, and that restoring SAMe rescues neural tube development in vivo. Indeed, our recent experiments showed that SAMe prevented brain oxidative/nitrosative stress and neural tube defects (NTDs) induced by VPA in ICR mouse embryos. Although most studies alongside our hypothesis are experimental and the entire causal pathway in humans remains to be established, increased oxidative stress, reduced SAMe, and reduced SAMe:SAH ratio were also described in human NTDs.

## 1. Introduction

Neural tube closure is a highly coordinated developmental process that requires precise regulation of signaling, cell movements, and epigenetic control [1,2]. Abnormal neurulation produces NTDs, the second-most severe congenital malformations, with substantial morbidity driven by redox–epigenetic crosstalk [3,4,5]. While genetic and environmental risk factors for NTDs are established, the mechanistic links between extracellular stressors and defective neurulation remain incompletely defined [6,7].

Oxidative/nitrosative stress represents a major upstream driver of NTDs (and various neurodevelopmental disorders) through redox–epigenetic crosstalk [8]. During neurulation, DNA and histone methylation orchestrate gene expression changes essential for neural plate morphogenesis and neural crest specification [9,10]. These epigenetic processes largely depend on SAMe, the universal methyl donor synthesized through one-carbon metabolism [11]. Critically, oxidative stress diverts SAMe toward glutathione (GSH) synthesis to combat reactive oxygen/nitrogen species, thereby limiting methyl donor availability and destabilizing epigenetic regulation [12,13,14,15]. SAMe depletion and resultant hypomethylation have been directly implicated in NTDs pathogenesis [16,17].

Environmental teratogens exemplify this redox–epigenetic axis. VPA and maternal diabetes elevate reactive oxygen species, suppress SAMe-dependent methylation, and disrupt neural tube closure through shared downstream pathways including Wnt/β-catenin inhibition and neuroepithelial apoptosis [18,19,20,21,22,23]. Despite this convergence, SAMe has received surprisingly little attention as a potential mechanistic nexus between redox imbalance and epigenetic dysregulation during neurulation.

This review aims to elucidate how redox stress promotes NTDs through SAMe depletion and epigenetic disruption. We synthesize evidence linking redox signaling, one-carbon metabolism, and epigenetic dynamics during neural tube formation, using VPA teratogenicity and diabetic embryopathy as exemplars. We further develop this framework by presenting VPA-induced exencephaly, including our SAMe rescue study in ICR mice, as proof of concept for a shared redox–epigenetic mechanism in neurulation. By examining this redox–epigenetic crosstalk, we posit SAMe insufficiency as a mechanistic node and potential preventive and therapeutic target in NTD research.

## 2. Literature Search Strategy

A comprehensive, non-systematic literature search was conducted for this conceptual narrative review between August 2025 and June 2026 using PubMed and Google Scholar. The search prioritized English-language, peer-reviewed original mechanistic studies, clinical or human observational studies, and recent high-quality review articles relevant to NTDs, redox biology, one-carbon metabolism, epigenetic regulation, and SAMe. More recent publications were preferentially considered; however, seminal earlier studies were retained when necessary to establish foundational mechanisms. Reference lists of eligible original articles and relevant reviews were also manually screened to identify additional pertinent studies. Searches used combinations of terms related to NTDs and neurulation; epigenetic regulation; oxidative and nitrosative stress; one-carbon metabolism and SAMe; and relevant experimental models or exposures. Representative search terms included: “neural tube defects” OR “NTDs” OR “neurulation” OR “neural tube closure”; “NTDs and epigenetics” OR “epigenetic basis of NTDs” OR “DNA methylation and NTDs” OR “histone modification and NTDs”; “oxidative stress and NTDs” OR “nitrosative stress and NTDs” OR “oxidative stress, epigenetics and NTDs”; “SAMe and NTDs” OR “S-adenosylmethionine and neurulation” OR “SAMe, oxidative stress and NTDs” OR “one-carbon metabolism and NTDs”; “valproic acid and NTDs” OR “diabetic embryopathy and NTDs”; and “SAMe–NTD–epigenetic crosstalk.” Studies were included when they addressed one or more of the following domains: redox or nitrosative stress during embryonic development; antioxidant defense; folate-dependent one-carbon or methionine metabolism; SAMe, S-adenosylhomocysteine (SAH), homocysteine, or glutathione metabolism; DNA, histone, or RNA epigenetic regulation; developmental signaling relevant to neurulation; or experimental and clinical evidence of NTD pathogenesis. Animal and embryo-based mechanistic studies were prioritized because they provide direct relevance to neurulation, while cell-based and human studies were included when they contributed mechanistic, biochemical, epigenetic, or clinical evidence. Studies were excluded when they were not relevant to neural development, NTDs, redox biology, one-carbon metabolism, or epigenetic regulation. Duplicate records and non-peer-reviewed sources were not included. Because this was a conceptual narrative review, the search was not intended to be exhaustive or systematic. No formal review protocol was registered, no PRISMA-based screening process or validated risk-of-bias tool was applied, and no meta-analysis or quantitative evidence synthesis was performed. Instead, evidence was interpreted qualitatively according to its mechanistic relevance, experimental context, and contribution to the proposed redox–one-carbon–epigenetic framework for NTD pathogenesis.

## 3. Brief Molecular Overview of Neural Tube Closure and Defects

During pregnancy, normal neural tube closure depends on tightly regulated signaling pathways that coordinate molecular and physiological processes with dynamic cellular rearrangements. These events drive lateral cell movements toward the dorsal midline to narrow the germ layers, while dorsal midline cells extend along the anteroposterior (AP) axis to elongate the embryo [24]. Major signaling pathways implicated in neural tube formation include Wnt and sonic hedgehog, fibroblast growth factor, Notch, and retinoic acid. The precise temporal and spatial integration of these pathways is essential for successful neurulation. Consequently, their abnormal regulation or interaction can result in NTDs and other neurodevelopmental disorders (NDDs) [25,26,27,28,29,30,31,32,33,34]. These signaling pathways also interact with broader molecular, genetic, and epigenetic processes, particularly chromatin remodeling and methylation dynamics, to establish the coordinated developmental “rhythm” required for proper neural tube formation [35].

Oxidative stress has been shown to disrupt several of these pathways, including Wnt/β-catenin and sonic hedgehog, primarily through cellular damage and apoptosis, thereby impairing neural tube closure [36,37,38]. These highlight the critical interplay between oxidative stress and epigenetic regulation in the etiology of NTDs and underscore important avenues for therapeutic intervention and future research.

## 4. Epigenetic Basis of Neural Tube Defects

The human epigenome comprises several interrelated regulatory layers, including DNA methylation, histone modifications (e.g., methylation and acetylation), and non-coding RNAs, which together are essential for normal cellular function. These coordinated mechanisms form a dynamic epigenetic machinery that precisely fine-tunes gene expression in space and time. Dysregulation of this machinery has been implicated in multiple developmental disorders, including NTDs [17]. Epigenetic involvement in NTDs is supported by observations of transgenerational neural tube and other congenital defects in offspring of individuals exposed in utero to VPA [39]. Aberrant DNA or histone hypermethylation and hypomethylation of neurulation-related genes have been associated with defective neural tube closure, underscoring the importance of tightly controlled methylation during early development [9]. Epigenetic alterations influence core cellular processes, including proliferation, differentiation, apoptosis, and autophagy, that are critical for neural tube morphogenesis [3]. Accordingly, understanding how specific epigenetic marks regulate gene expression during neurulation is essential for interpreting gene expression changes in developmental disorder contexts. This section focuses on DNA methylation and histone acetylation, the two epigenetic marks most extensively studied in NTD research.

### 4.1. DNA Methylation Mechanism Related to Neural Tube Defects

**Figure 1:**
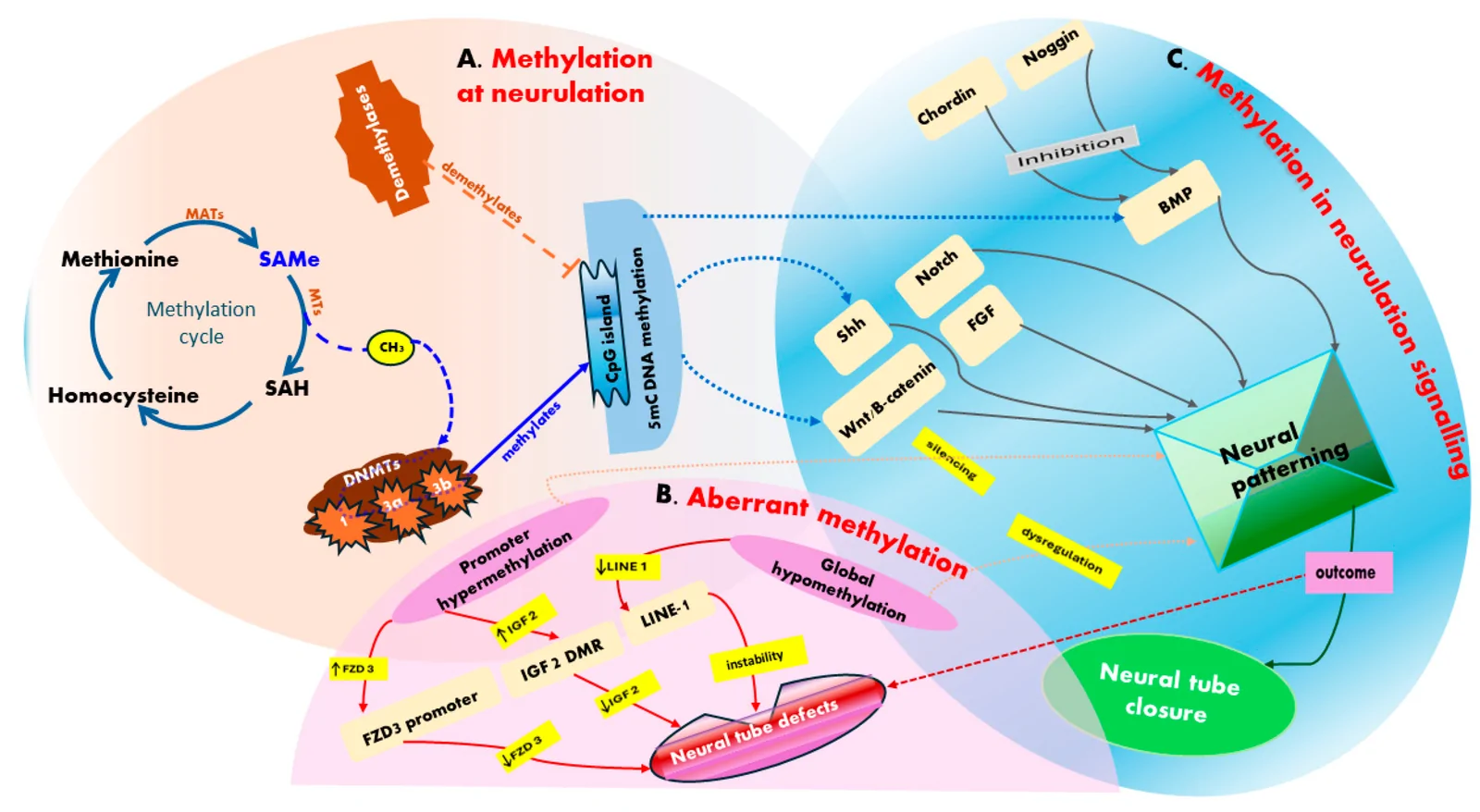
DNA methylation dynamics during neural tube development and their disruption in neural tube defects (NTDs). Figure 1 illustrates DNA methylation–dependent regulation of gene expression during neurulation and its potential contribution to NTD pathogenesis. In the methylation cycle, methionine is converted to SAMe, the methyl-group donor, and subsequently to SAH and homocysteine. SAM donates –CH_3_ through DNMTs. DNMT3A and DNMT3B establish de novo DNA methylation, whereas DNMT1 maintains methylation patterns during DNA replication. During normal neurulation, methylation-dependent regulation of developmental signaling pathways, including BMP signaling and its antagonists chordin and noggin, as well as Wnt/β-catenin, Shh, Notch, and FGF signaling, contributes to neural plate elevation, fusion, patterning, and neural tube closure. (**A**) illustrates the role of physiological DNA methylation during neural tube development. (**B**) depicts aberrant methylation, including promoter hypermethylation, global hypomethylation, and altered methylation of IGF2 differentially methylated region (DMR) and long interspersed nuclear element 1 (LINE-1). These changes may contribute to genomic instability, altered developmental gene expression, and abnormal neural crest specification or migration. (**C**) summarizes the relationship between methylation-dependent regulation of neurulation signaling, neural patterning, and neural tube closure. Disruption of DNMT activity or one-carbon metabolism may therefore disturb the balance between DNA methylation and demethylation, impair neural crest development, and result in NTDs. Dashed arrows indicate indirect or proposed relationships.

DNA methylation (Figure 1) involves the covalent addition of a methyl group (–CH_3_) to the 5-carbon of cytosine, most commonly within CpG dinucleotides in gene regulatory regions, where it modulates chromatin accessibility and transcriptional activity. This reaction is catalyzed by DNA methyltransferases (DNMTs) using SAMe as the methyl donor [40]. DNA and histone methylation states are balanced via dynamically coordinated methylation and demethylation [41]. During neurulation, these dynamics control transcriptional programs governing neural plate specification, neural crest formation, and neuroepithelial differentiation. Disruption of this dynamic methylation-demethylation balance has been causally linked to defective neural tube closure [3,42].

DNMTs function through the transmethylation pathway and have distinct catalytic and regulatory roles in development [43]. Methyltransferases (DNMT3A and DNMT3B) are required for establishing embryonic methylation patterns and maintaining chromatin stability during neural tube formation [44]. Conditional deletion of *Dnmt3a* or *Dnmt3b* in neural tissues leads to abnormal neural tube closure, with *Dnmt3b* loss preferentially causing rostral NTDs and neural crest defects, while *Dnmt3a* disruption alters neural gene expression boundaries and morphogenesis [45,46,47].

A defining feature of DNA methylation abnormalities in NTDs is loss of methylation fidelity rather than a uniform pattern of hypermethylation or hypomethylation. Because neurulation requires precise spatiotemporal control of developmental genes, both excessive methylation and insufficient methylation can disrupt signaling, proliferation, differentiation, and morphogenesis [40,48]. Thus, successful neural tube closure depends on preserving methylation fidelity, maintaining balanced, locus-appropriate methylation, rather than enforcing a specific methylation state at any single locus [49].

### 4.2. Histone Modification Marks in the Etiology of Neural Tube Defect

Histone modifications constitute a core layer of epigenetic regulation that controls chromatin accessibility and transcription during embryogenesis. These marks are dynamically regulated by opposing enzyme systems, including histone acetyltransferases (HATs) and histone deacetylases (HDACs), as well as histone methyltransferases and demethylases, which together shape developmental gene expression programs. In general, histone acetylation is associated with transcriptional activation via loose chromatin, whereas deacetylation promotes chromatin compaction and transcriptional repression [50,51]. Through the combinatorial “histone code,” post-translational modifications integrate developmental signals into precise spatiotemporal transcriptional outputs that regulate cell proliferation, differentiation, and tissue morphogenesis [52,53]. Disruption of these processes may contribute to developmental abnormalities and congenital malformations [54,55]. Accumulating evidence highlights altered histone acetylation as a key determinant of interrupted neural tube closure and neural crest development (Table 1).

#### Histone Acetylation and Deacetylation in Neural Tube Defects

**Figure 2:**
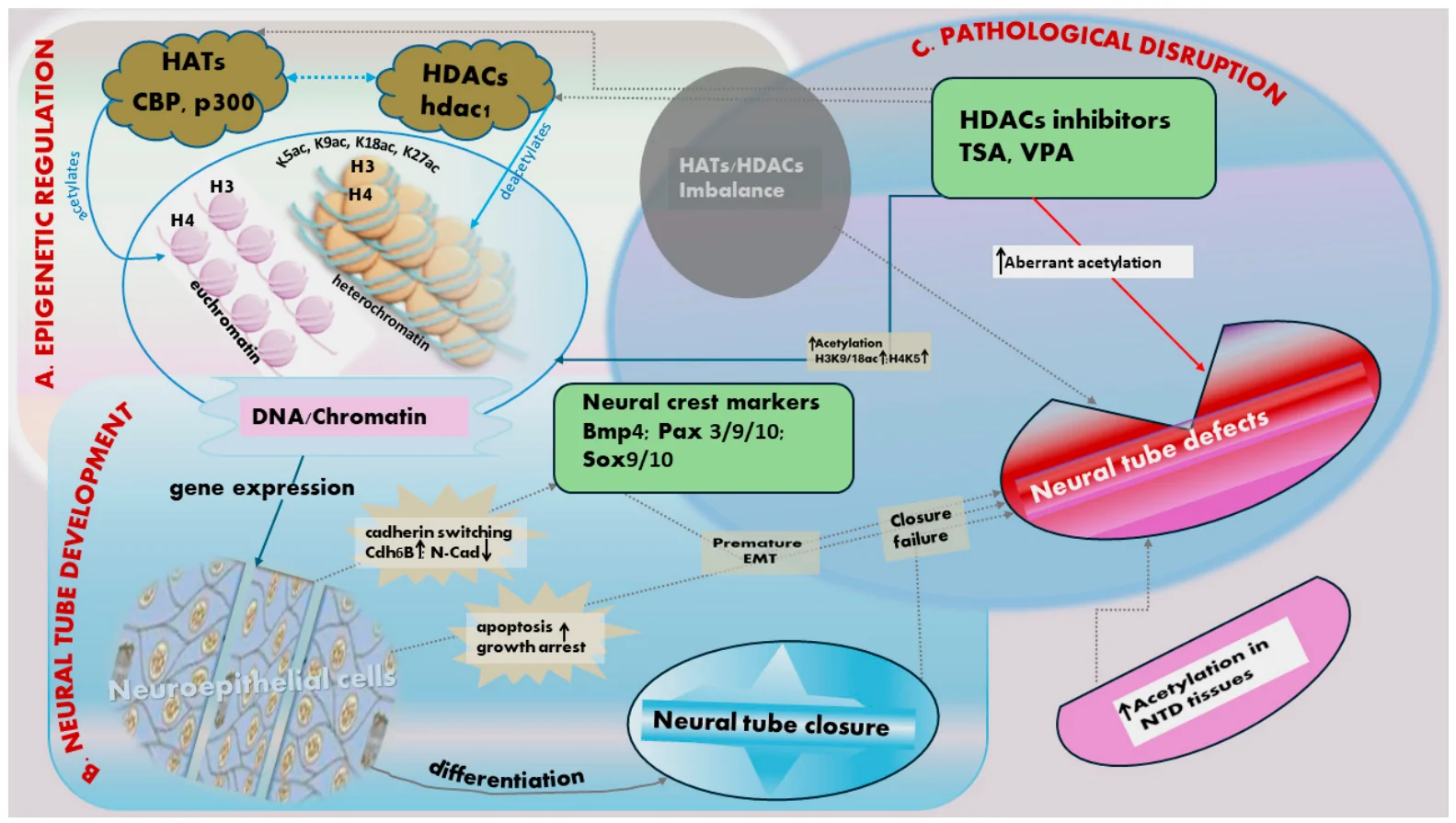
Schematic representation of histone acetylation dynamics during neural tube development and their role in neural tube defects (NTDs). This schematic depicts the opposing actions of histone acetyltransferases (HATs) and histone deacetylases (HDACs). (**A**) How histone acetyltransferases (HATs; CBP/p300) and histone deacetylases (HDACs; including HDAC1) oppositely regulate histone H3/H4 acetylation and chromatin accessibility. HAT-mediated acetylation of lysine residues, including H3K9ac, H3K18ac, H3K27ac, and H4K5ac, is associated with chromatin relaxation and transcriptional activation, whereas HDAC-mediated deacetylation promotes chromatin compaction. (**B**) During neurulation, acetylation-dependent gene expression in neuroepithelial cells supports proliferation, differentiation, neural crest specification, and neural tube closure. Neural crest-associated markers include bone morphogenetic protein 4 (Bmp4), paired box genes Pax3/9/10, and SRY-box transcription factors Sox9/10. Regulated cadherin switching, characterized by increased cadherin 6B (Cdh6B) and reduced N-cadherin, is also depicted. (**C**) Pharmacological HDAC inhibition by TSA or VPA disrupts the HAT–HDAC balance and promotes aberrant hyperacetylation. This dysregulation may alter developmental gene expression, induce premature mesenchymal transition, apoptosis, growth arrest, and impair neural tube closure. Increased H3K9/18 acetylation and H4K5 acetylation are indicated as acetylation changes associated with NTD tissues. Dashed arrows indicate indirect or proposed relationships.

When DNA methylation fidelity is compromised, developmental gene expression, cell fate specification, proliferation, and morphogenesis are impaired, underscoring the essential epigenetic dimension of neurulation. Excessive or insufficient histone acetylation (Figure 2), along with altered histone methylation, can similarly disrupt the epigenetic programs required for normal neural tube formation [56,57,58,59]. Histone acetylation regulates chromatin accessibility and transcriptional competence during early development by modulating lysine acetylation on histone tails [56,57,58,59,60]. Converging evidence from human tissues, genetic models, and teratogen exposure indicates that balanced acetylation dynamics are required for successful neural tube closure [17,56,57,58,59,61,62].

Experimental studies provide direct support for this model. Local HDAC inhibition with trichostatin A (TSA) in chick embryos caused posterior neuropore closure defects that coincided with increased H3/H4 acetylation, neuroepithelial apoptosis, and aberrant cadherin switching (upregulated cadherin 6B, downregulated N-cadherin), together with premature upregulation of neural crest markers (*Bmp4*, *Pax3*, *Sox9*, *Sox10*), placing HDAC activity upstream of transcriptional and morphogenetic events required for neurulation [58]. Similarly, VPA, a clinically relevant HDAC inhibitor, induces histone hyperacetylation, cell-cycle arrest, and apoptosis in embryonic neural progenitors and downregulates developmental regulators (e.g., *Folr1*, *Pax3*), linking acetylation perturbation to broad transcriptional reprogramming during neurulation [5,62,63,64]. In human tissue, increased histone acetylation (including elevated H4K5ac) has been reported in brains from NTDs cases relative to controls, implicating altered acetylation marks in human disease [59].

Genetic data reinforce this view: loss of Gcn5 HAT activity disrupts cranial neural tube closure and causes exencephaly in mice, demonstrating that intact HAT function is indispensable for neural tube formation [61]. Beyond acetylation, histone methylation contributes to NTD pathogenesis via patterning genes: in a retinoic acid–induced mouse model, altered H3K27me3 enrichment and dysregulated *Hox* gene expression were observed across neurulation stages, with human anencephaly samples showing abnormal H3K27me3 levels and inverse correlations with *HOXB4*, *HOXC4*, and *HOXD1* expression [65]. Genome-wide profiling further identified hundreds of differentially expressed genes in NTDs associated with H3K27me3 and H3K27ac remodeling, underscoring broad histone-mark changes during neural tube closure [59].

**Table 1:** Integrated evidence linking epigenetic dysregulation to neural tube defects.

Authors	Mechanistic Domain	Integrated Finding	Experimental Context
[40]	SAMe-dependent methylation	Inhibition of SAMe synthesis during early embryogenesis disrupts methyltransferase activity and neural tube closure.	Non-mutant, random-bred CD1 mouse fetal brain; tandem mass spectrometry and immunostaining
[44]	DNA methylation machinery	DNMT3A and DNMT3B are required for establishing embryonic methylation patterns and maintaining chromatin stability during neurulation.	Neuroepithelial cells prepared from pregnant ICR mice; immunofluorescence, luciferase assay, and electrophoretic mobility shift assay
[49]	Locus-specific DNA methylation	Increased methylation of the imprinted IGF2 locus in NTD tissue is inversely associated with IGF2 expression.	Brain tissue from stillborn fetuses; CpG methylation quantified by matrix-assisted laser desorption/ionization time-of-flight mass spectrometry
[48]	Global DNA methylation	Global DNA hypomethylation in fetal brain tissue is associated with increased NTD risk and severity.	Medically aborted NTD-affected fetal brain and skin; global DNA methylation analysis
[66]	Promoter methylation	Hypermethylation of the FZD3 promoter, rather than genetic polymorphism, is associated with spina bifida.	Stillborn fetal tissue and 5-azacytidine-treated HeLa cells; MassARRAY EpiTYPER analysis
[67]	Repetitive-element methylation	Hypomethylation of LINE-1 and genomic DNA is associated with increased NTD risk.	Stillborn neural tissue; LINE-1 methylation and total homocysteine assessment
[58]	Histone acetylation and HDAC activity	Trichostatin A-induced HDAC inhibition increases H3/H4 acetylation, disrupts cadherin switching, and promotes apoptosis during neurulation.	Chick embryos exposed to TSA in ovo; pharmacological inhibition of HDAC activity
[62]	VPA-induced histone hyperacetylation	Abnormal histone acetylation and HDAC inhibition induce cell-cycle arrest and apoptosis in VPA-exposed embryos.	Pregnant CD mice treated with VPA or TSA on gestational day 8; embryo explantation and histone-acetylation analysis
[59]	Histone acetylation in human NTDs	Human NTD brain tissue exhibits increased histone acetylation, including elevated H4K5ac, compared with controls.	Human NTD tissue; LC–ESI–MS, nano-HPLC–MS/MS, and Western blotting
[61]	Histone acetyltransferase function	Loss of Gcn5 histone acetyltransferase activity disrupts cranial neural tube closure.	Gcn5 HAT-mutant mice; molecular and developmental analyses
[65]	Histone methylation and patterning	Retinoic-acid-induced NTDs are associated with altered H3K27me3 enrichment and dysregulated Hox gene expression.	RA-induced mouse model examined at E8.5, E9.5, and E10.5; RNA sequencing and ChIP sequencing
[68]	SAMe-dependent methylation	Inhibition of SAMe synthesis during early embryogenesis disrupts methyltransferase activity and neural tube closure.	Non-mutant, random-bred CD1 mouse fetal brain; tandem mass spectrometry and immunostaining
[69]	DNA methylation machinery	DNMT3A and DNMT3B are required for establishing embryonic methylation patterns and maintaining chromatin stability during neurulation.	Neuroepithelial cells prepared from pregnant ICR mice; immunofluorescence, luciferase assay, and electrophoretic mobility shift assay

Studies are grouped according to the principal epigenetic mechanism or biological outcome they address, including DNA methylation, histone modification, SAMe-dependent regulation, RNA methylation, developmental signaling, and neuroepithelial responses. Abbreviations: BaP, benzo[a]pyrene; ChIP, chromatin immunoprecipitation; CUT&Tag, cleavage under targets and tagmentation; DNMT, DNA methyltransferase; E, embryonic day; FZD3, frizzled class receptor 3; Gcn5, general control non-repressed protein 5; H3K27ac, histone H3 lysine 27 acetylation; H3K27me3, histone H3 lysine 27 trimethylation; H4K5ac, histone H4 lysine 5 acetylation; HAT, histone acetyltransferase; HDAC, histone deacetylase; IGF2, insulin-like growth factor 2; ICR, Institute of Cancer Research mouse strain; LC–ESI–MS, liquid chromatography–electrospray ionization–mass spectrometry; LINE-1, long interspersed nuclear element 1; m6A, N6-methyladenosine; NTDs, neural tube defects; RA, retinoic acid; SAMe, S-adenosyl-L-methionine; TSA, trichostatin A; VPA, valproic acid; Wnt, Wnt signaling; β-catenin, beta-catenin.

Findings from amniotic-fluid stem cells of anencephalic fetuses show increased H3K9ac and H3K18ac, whereas spinal cord tissues from NTD patients exhibit reduced H3K4ac at *WNT2B* and *WNT7B* promoters, indicating that acetylation changes are both tissue- and locus-specific and can modulate Wnt signaling relevant to neurulation [18]. In retinoic acid–induced NTDs models, altered apoptosis-related gene expression accompanies reduced H3K27ac at TNF-associated promoters, and mice lacking CBP or P300 or homozygous Gcn5 show embryonic lethality and cranial NTDs, demonstrating that HAT maintenance is indispensable for neural tube formation [17]. In summary, converging evidence from human tissues, genetic models, and teratogen exposure studies demonstrate that both excessive and insufficient histone acetylation, along with altered histone methylation, can disrupt the epigenetic programs required for normal neural tube formation. These findings strongly position histone modification dysregulation as a fundamental driver of NTD pathogenesis.

## 5. Redox Stress in Neural Tube Defects

**Figure 3:**
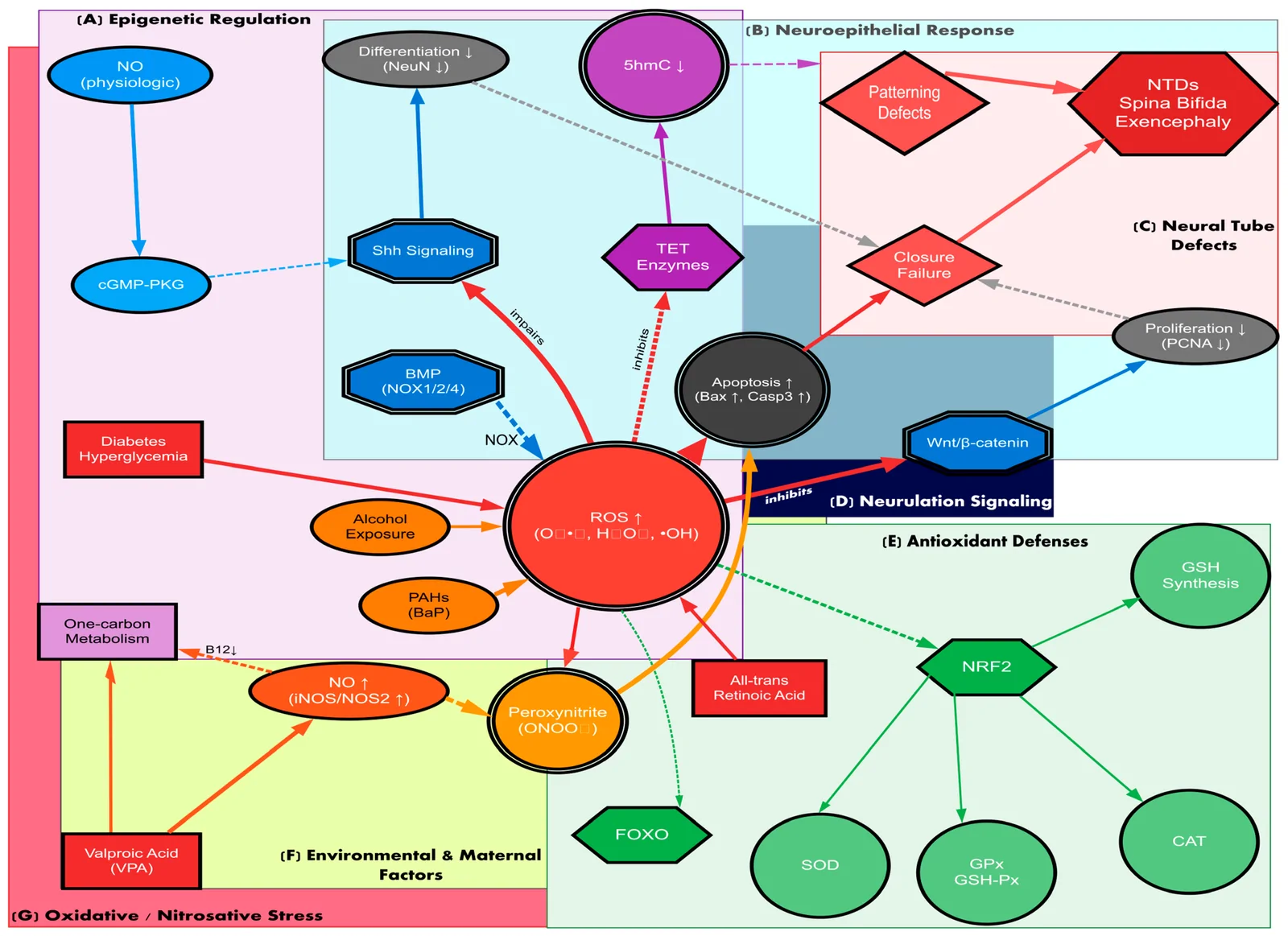
Schematic representation of oxidative and nitrosative stress mechanisms disrupting neurulation and driving neural tube defects (NTDs). This integrated model illustrates how redox imbalance, signaling pathway inhibition, epigenetic dysregulation, and apoptosis converge to drive NTD pathogenesis during early embryogenesis. Environmental toxicants (PAHs, VPA, all-trans retinoic acid) and maternal metabolic stressors (diabetes, alcohol) trigger excessive production of ROS (O_2_^−^, H_2_O_2_, –OH) and RNS (**F**), forming a central redox hub in which peroxynitrite (ONOO^−^) is generated through superoxide–NO interactions (**G**). This oxidative burden overwhelms NRF2/FOXO-mediated antioxidant defenses, including SOD, GPx, CAT, and GSH, leading to persistent redox imbalance (**E**). Elevated ROS directly inhibits critical neurulation signaling pathways (**D**), suppressing Wnt/β catenin dependent proliferation and convergent extension, impairing Shh-mediated anterior–posterior and dorsal–ventral patterning, and is further amplified by BMP-induced NOX1/2/4 activation, while physiological NO–cGMP supports sonic hedgehog signaling and increased iNOS activity shifts NO output toward nitrosative damage. Epigenetic disruption arises through ROS-mediated suppression of TET enzymes, 5hmC levels required for chromatin remodeling, and through NO-dependent B12 sequestration that impairs one-carbon metabolism (**A**). These converging insults compromise neuroepithelial integrity (**B**), manifesting as reduced proliferation (PCNA↓), impaired differentiation (NeuN↓), and apoptosis (Bax↑, caspase 3↑), all of which are essential for neural fold elevation and fusion. The downstream consequences are patterning defects and closure failure, culminating in NTDs including spina bifida and anencephaly (**C**). Red arrows indicate inhibitory or disruptive effects; dashed lines denote activation, regulation, or feedback loops.

Reactive oxygen species (ROS) and reactive nitrogen species (RNS) are essential in several metabolic reactions throughout prenatal and postnatal life. If they exceed physiological levels, oxidative and/or nitrosative stress occurs, with possible resulting serious metabolic disturbances [70]. Although ROS and RNS can participate in regulated developmental signaling, our focus is on the transition from controlled redox activity to pathological oxidative/nitrosative stress during embryonic insult. When environmental or metabolic stressors increase ROS/RNS production beyond the buffering capacity of the neurulating embryo (Figure 3), redox signaling becomes persistent and dysregulated, promoting molecular injury rather than an adaptive developmental response [71,72,73,74]. Increased demand for glutathione and other antioxidant defenses can shift homocysteine metabolism toward transsulfuration and cysteine/glutathione production, thereby reducing flux through remethylation and limiting methionine regeneration. This may lower SAMe availability, increase the relative influence of SAH-mediated methyltransferase inhibition, and reduce cellular methylation potential [75,76,77]. In support of this redox–methylation connection, homocysteine exposure in chick embryos has been associated with increased oxidative species, reduced antioxidant enzyme activity, decreased SAMe/SAH, altered DNA methyltransferase expression, DNA hypomethylation, and NTD formation; these effects were attenuated by N-acetylcysteine and choline. Redox stress may also directly perturb DNMTs, TET enzymes, and histone-modifying proteins [78,79,80]. Such redox-driven epigenetic disruption can compromise DNA methylation fidelity, thereby altering developmental gene expression, cell-fate specification, proliferation, and morphogenesis. Oxidative and nitrosative stress can erode the fidelity of embryonic epigenetic machinery by diverting one-carbon metabolism toward antioxidant defense and direct perturbation of redox-sensitive signaling during neurulation [71,72,73,74]. Environmental and biological stressors that increase ROS/RNS generation activate signaling cascades that disrupt nuclear and cytoplasmic processes during neurulation, positioning redox stress as an active driver of altered developmental signaling [73,74].

During early embryogenesis, the embryo has limited antioxidant buffering capacity [71], and oxidative stress may disrupt signaling pathways required for neural tube closure (Figure 3). This suppresses Wnt/β-catenin activity during anterior–posterior patterning, impairing proliferation, differentiation, and barrier integrity. Conversely, Wnt activation can counteract oxidative stress and restore these processes [81,82]. Oxidative stress also impairs sonic hedgehog signaling by disrupting Shh processing, secretion, and downstream effectors. Elevated ROS in maternal diabetes and alcohol exposure alter *Shh* expression and anterior–posterior patterning, compromising neural tube closure [83,84]. Bone morphogenic protein signaling induces NADPH oxidases (NOX1/2/4) to generate ROS, and ROS in turn modulates BMP expression and receptor activity. Imbalance in this loop alters cell shape, differentiation, and dorsal-ventral patterning via Smad-dependent and Smad-independent pathways, ultimately interfering with neural tube closure [83,84,85,86,87].

In human in neural tissue, from 92 terminated NTD cases and 33 terminated non-malformed fetuses, global 5-hydroxymethylcytosine (5hmC) was reported lower in NTDs cases than controls, and increased ROS was associated with 5hmC loss, implicating PAH-induced oxidative stress in NTD pathogenesis [88]. Because 5hmC supports gene regulation, chromatin organization, and neural development, its ROS-mediated depletion provides a plausible epigenetic mechanism linking oxidative stress to NTD risk [88]. A similar pattern was observed in an all-trans retinoic acid (atRA) rat model: malformed spinal cords showed a pro-oxidant state (reduced SOD/GSH-Px, increased MDA), neuroepithelial loss, impaired differentiation/proliferation (reduced NeuN/PCNA), and mitochondrial apoptosis (altered BAX/BCL-2, increased cleaved caspase 3), directly linking redox imbalance to defect formation [89,90]. This injury is intensified by nitrosative stress, which cooperates with oxidative pathways to drive peroxynitrite-mediated apoptosis, whereas antioxidant enzymes such as SOD protect against this damage, hence reducing NTD incidence [91,92].

**Table 2:** Mechanistic evidence linking oxidative and nitrosative stress to neural tube defects.

Authors	Mechanistic Domain	Integrated Finding	Experimental Design
[73,74]	Redox burden	Environmental and biological stressors overwhelm embryonic antioxidant defenses and increase ROS/RNS	Cellular model
[81,82]	Wnt signaling	Oxidative stress inhibits Wnt/β-catenin signaling and impairs proliferation and convergent extension	Mechanistic review
[84]	Shh signaling	Elevated ROS disrupts Shh processing and patterning	Chicken embryo
[86,87]	BMP–NOX crosstalk	BMP induces NOX-dependent ROS, while ROS feeds back on BMP signaling	ES-cell differentiation model
[88]	Epigenetic regulation	Increased ROS is associated with loss of 5hmC in NTD tissue	Human fetal tissue and embryo culture
[89]	Antioxidant failure and apoptosis	Reduced SOD/GPx and increased lipid peroxidation accompany neuroepithelial apoptosis	atRA rat model
[91,92]	Nitrosative stress	Excess NO and peroxynitrite promote apoptosis and NTDs	Diabetic mouse model
[93]	VPA-induced nitrosative stress	VPA increases iNOS, nitration, S-nitrosylation, and apoptosis	ICR mouse embryo
[94]	NO-dependent protection	Modulation of NO signaling supports neurulation under VPA exposure	ICR mouse model
[95,96,97]	Antioxidant rescue	Vitamins C/E and nitroxide radicals reduce oxidative stress and NTD incidence	Diabetic and embryo-culture models
[98,99]	One-carbon/redox interface	Reduced SAMe and altered B12-dependent metabolism accompany diabetic embryopathy	Diabetic models

Studies are grouped according to the principal mechanistic domain, integrated biological finding, and experimental context. Abbreviations: atRA, all-trans retinoic acid; B12, vitamin B12; BMP, bone morphogenetic protein; ES, embryonic stem; GPx, glutathione peroxidase; ICR, Institute of Cancer Research mouse strain; iNOS, inducible nitric oxide synthase; NTDs, neural tube defects; NO, nitric oxide; NOX, NADPH oxidase; RNS, reactive nitrogen species; ROS, reactive oxygen species; SAMe, S-adenosyl-L-methionine; Shh, sonic hedgehog; SOD, superoxide dismutase; VPA, valproic acid; Wnt, Wnt signaling; β-catenin, beta-catenin; 5hmC, 5-hydroxymethylcytosine.

Physiological NO signaling supports neural tube formation, whereas excess NO or a disturbed NO–ROS balance promotes oxidative/nitrosative stress and increases NTD risk [100]. In diabetes-related neurulation defects, lower NO may be protective against apoptosis, whereas higher NO can react with superoxide to form peroxynitrite. While NO can further modulate differentiation via the guanylate cyclase–cGMP axis and influence Shh-related programs, NO-related metabolites sequester vitamin B12 to cause deficiency that perhaps impairs one-carbon metabolism and methylation capacity [75,76,77]. Interventions that preserve properly regulated NO signaling (e.g., sildenafil citrate) have been reported to support neurulation and reduce teratogenic outcomes [94]. Overall (Table 2), the evidence indicates that elevated oxidative/nitrosative stress beyond physiological thresholds during neurulation overwhelms embryonic antioxidant defenses, disrupts Wnt, Shh, and BMP signaling, and promotes peroxynitrite-mediated neuroepithelial apoptosis. By increasing glutathione demand and impairing one-carbon metabolism, redox imbalance may reduce SAMe availability and methylation capacity, contributing to epigenetic dysregulation, impaired neural tube closure, and NTDs [72,88,89,90].

## 6. Folate-Dependent One-Carbon Metabolism as an Upstream Regulator of SAMe and Epigenetic Stability During Neurulation

Folate-dependent one-carbon metabolism is an upstream regulator of neurulation because it supplies one-carbon units for nucleotide synthesis, redox homeostasis, methionine regeneration, and production of SAMe. In the rapidly proliferating neuroepithelium, sustained SAMe availability is required to preserve methylation fidelity across DNA, histone, and RNA substrates and thereby maintain transcriptional programs governing neural plate specification, neural fold elevation, and closure [40,101,102].

Mitochondrial and cytosolic one-carbon pathways cooperate to sustain this methylation capacity. Mitochondrial enzymes, including SHMT2, MTHFD2, and MTHFD1L, generate formate from serine- and glycine-derived one-carbon units; formate is then exported to the cytosol, where it supports folate-dependent biosynthesis and methionine/SAMe regeneration [101]. The developmental importance of this mitochondrial supply is illustrated by *Mthfd1l*-deficient mice, which develop highly penetrant neural tube defects despite adequate dietary folate; maternal formate supplementation reduces NTD incidence and partially rescues embryonic growth, demonstrating that mitochondrial one-carbon flux is indispensable during neurulation [103,104].

Disruption of folate-dependent one-carbon metabolism, through nutritional deficiency, genetic variation, or redox-driven metabolic stress, reduces SAMe availability, increases SAH, and lowers methylation potential. Experimental inhibition of the methionine cycle by ethionine or cycloleucine increases NTD incidence, directly demonstrating that an intact methylation cycle is essential for neural tube closure [40]. In parallel, the *MTHFR* C677T variant, which reduces generation of 5-methyltetrahydrofolate required for methionine regeneration, has been associated with NTD risk in human studies [105].

The downstream consequence is not a defect in one epigenetic mark or gene, but a loss of epigenetic fidelity. Folate deficiency and SAMe depletion alter DNA methylation and hydroxymethylation, histone modifications, RNA $m^{6}A$ methylation, imprinting, and protein methylation, thereby disrupting developmental pathways such as Wnt/β-catenin and sonic hedgehog signaling [69,106,107,108,109,110]. For example, ethionine-mediated SAMe depletion altered neural progenitor differentiation and NTD formation [69].

Collectively, these findings support a unified model in which folate-dependent one-carbon metabolism governs neurulation by maintaining SAMe-dependent methylation capacity. This provides a mechanistic link between folate status, redox stress, chromatin dysregulation, and NTD pathogenesis [40,101,102].

## 7. S-Adenosylmethionine as a Crucial Metabolic and Epigenetic Regulator of Neurulation

During embryonic development, SAMe occupies a critical position at the interface of one-carbon metabolism, epigenetic regulation, and antioxidant defense, all of which must be tightly coordinated for normal neurulation. SAMe is synthesized from methionine and ATP by methionine adenosyltransferase (MAT), and after methyl donation it is converted to S-adenosylhomocysteine (SAH). Because SAH is a potent inhibitor of methyltransferases, the SAMe:SAH ratio is a key determinant of cellular methylation potential. Homocysteine is then either methylated to methionine through folate- and cobalamin-dependent pathways or directed into the transsulfuration pathway to support glutathione synthesis. Even modest reductions in this ratio can impair methylation, alter chromatin-dependent gene regulation, and weaken redox buffering capacity-conditions that are especially harmful during neural tube formation.

SAMe integrates transmethylation, transsulfuration, and polyamine synthesis, allowing it to coordinate epigenetic programming with antioxidant defense [14]. By activating cystathionine β-synthase, SAMe promotes transsulfuration and supports cysteine and GSH production. Under oxidative stress, increased GSH demand diverts homocysteine away from remethylation, reducing methionine regeneration and depleting SAMe pools. This redox-driven shift links oxidative stress to reduced methyl donor availability and chromatin dysregulation during neurulation. Evidence from non-developmental disease models reinforces the consequences of SAMe depletion: chronic ethanol exposure lowers SAMe and promotes liver injury [111], while *Mat1a*-deficient mice show SAMe deficiency, reduced GSH, mitochondrial dysfunction and oxidative stress, all of which are improved by SAMe supplementation [112,113,114,115,116,117,118,119]. Together, these studies show that SAMe insufficiency can destabilize both redox homeostasis and epigenetic regulation [112,113,114,115,116,117,118,119].

Accumulating evidence identifies SAMe as a rate-limiting determinant of neurulation. Ref. [68] showed that cycloleucine-induced SAMe deficiency, through direct MAT inhibition, causes neural tube defects in mice and HT-22 cells by suppressing *Pax3* transcription and protein expression, destabilizing dorsal neuroepithelial identity and the proliferation–apoptosis balance. Mechanistically, SAMe deficiency reduces EdU incorporation, phospho-histone-H3 labeling, and PCNA expression, while increasing BAX and activating CASP3, leading to excessive neuroepithelial apoptosis and inadequate proliferative expansion required for neural fold elevation, bending, and fusion. Upregulation of *Pax3* rescues these phenotypes, restoring proliferation, suppressing apoptosis, and rescuing potential NTDs [68]. These findings make SAMe not merely a methyl donor, but a rate-limiting metabolic and epigenetic determinant that couples one-carbon flux, chromatin regulation, and redox-dependent cell-survival programs to neurulation.

Furthermore, studies show that SAMe regulates epigenetic mechanisms beyond DNA methylation. Ref. [69] reported that ethionine-mediated SAMe depletion, via MAT competition and a reduced SAMe:SAH ratio, causes neural tube defects by disrupting METTL3- and ALKBH5-dependent m6A RNA modification, suppressing Wnt/β-catenin signaling, lowering CyclinD1 and C-Myc expression, and perturbing neuroepithelial cell cycle progression and apoptosis. This positions SAMe as a critical node linking one-carbon flux, RNA epigenetics, and canonical signaling to neural tube closure.

SAMe-centered evaluation of one-carbon metabolism further reinforces SAMe’s role in NTDs susceptibility. Folate deficiency reduces intracellular SAMe, promotes global DNA hypomethylation, and increases NTD risk [120,121,122,123]. Similarly, defects in glycine cleavage enzymes (AMT, GLDC) reduce one-carbon flux and SAMe-dependent methylation, contributing to NTDs with variable penetrance [103]. SAMe deficiency also affects fundamental cellular processes required for neural tube morphogenesis, including cell-cycle progression and apoptosis. SAMe limitation induces G1 arrest characterized by altered Cdk4/Cdk2 activity through p38-MAPK–MK2 signaling, independently of mTORC1 or polyamine metabolism [124]. Disruption of these proliferation programs compromises neuroepithelial expansion and bending dynamics essential for closure, reinforcing SAMe’s role as a master regulator of developmental timing and tissue morphogenesis [125,126].

Potentially, these findings position SAMe as a rate-limiting metabolic and epigenetic determinant of neurulation, integrating one-carbon metabolism, redox buffering, chromatin regulation, and developmental signaling into a unified framework for neural tube defect pathogenesis [40,68,69,127].

## 8. Valproic Acid and SAMe Depletion in Neural Tube Defect Pathogenesis

Valproic acid provides a well-defined example of how redox imbalance, epigenetic disruption, and one-carbon metabolic failure converge on SAMe insufficiency during neurulation. VPA (2-propylpentanoic acid) is a clinically relevant teratogen and HDI whose developmental toxicity is strongly stage-dependent [8]. In mice, exposure during neurulation (approximately embryonic days E8–E9) reliably produces neural tube defects (NTDs) [71]. Later exposure around E12 perturbs subsequent neurodevelopmental programs [128], whereas exposure during E13–E14, coincident with facial process fusion, predisposes embryos to cleft lip and palate [5,129,130,131]. As an HDI, VPA increases histone acetylation and disrupts chromatin states required for neural tube closure, leading to altered transcription, growth arrest, and apoptosis in neuroepithelial cells [62,64,132,133].

During early development, VPA-induced histone hyperacetylation has also been linked to replication-independent DNA demethylation and persistent transcriptional reprogramming [134]. However, chromatin acetylation alone does not fully explain the specificity or severity of VPA-induced neurulation defects. VPA also increases fetal redox burden through mitochondrial dysfunction and impaired antioxidant defenses [93,135,136,137]. Under these conditions, epigenetic regulation becomes metabolically constrained because DNA and histone methylation require sustained redox balance and methyl-donor availability, linking VPA-induced oxidative stress to SAMe-dependent epigenetic failure. A key component of this disturbance is dysregulated nitric oxide signaling [93,138].

Within this framework, the mechanistic importance of VPA-induced oxidative and nitrosative stress lies in its impact on one-carbon metabolism and methylation capacity. Studies in animal models of ASD show that perinatal SAMe and VPA exposure counteract each other’s effects in the brain [16,139,140,141].

VPA may further worsen this deficit by directly inhibiting MAT and impairing folate transport across placental and blood–brain barriers, thereby compounding redox-driven depletion of methyl-donor pools [142,143].The resulting loss of methylation potential destabilizes methylation-sensitive regulators of cell-cycle progression and apoptosis, including *p21*, *Bax*, and *Bcl-2*, and disrupts chromatin and transcriptional programs required for neural tube bending, hinge-point formation, and closure [16,139,140,141,144].

VPA teratogenicity acts as an epigenetic disruptor through HDAC inhibition while simultaneously amplifying oxidative and nitrosative stress and weakening antioxidant buffering capacity [5]. It therefore exemplifies how distinct teratogens can converge on a common SAMe-dependent metabolic bottleneck, offering a mechanistic explanation for overlapping NTDs phenotypes across diverse environmental and metabolic insults [68,69,127].

## 9. Redox–Epigenetic Interactions in Diabetic Embryopathy Converge on SAMe Insufficiency During Neurulation

Maternal diabetes represents a metabolically driven environment with negative effects on embryonic and fetal development. It illustrates how endogenous redox stressors converge on the same SAMe-dependent epigenetic vulnerability during neurulation. Hyperglycemia during early pregnancy exposes the developing embryo to sustained oxidative and nitrosative stress at a developmental stage characterized by limited antioxidant capacity and high epigenetic demand. As a result, diabetic embryopathy is strongly associated with NTDs, providing a clinically relevant model in which metabolic stress, rather than xenobiotic exposure, destabilizes neurulation through shared redox–epigenetic mechanisms [145,146,147].

In neurulating embryos, maternal hyperglycemia promotes excessive generation of ROS through glucose auto-oxidation, mitochondrial overload, and perturbed lipid and ketone metabolism. This redox imbalance is consistently accompanied by reduced antioxidant defenses, including SOD and CAT activity, impaired GSH redox potential, and elevated lipid peroxidation products such as malondialdehyde and 4-hydroxynonenal [148,149,150]. Reduced embryonic water- and lipid-soluble antioxidants were also observed by cyclic voltammetry [151]. Importantly, embryos that develop NTDs exhibit the most pronounced redox disturbances, whereas diabetic embryos without defects display oxidative indices closer to controls, underscoring oxidative stress as a proximal determinant of neurulation failure [152].

Hyperglycemia-induced redox stress activates conserved stress-response pathways that directly compromise neuroepithelial survival and morphogenesis. Oxidative signaling through ASK1–JNK–FOXO3A, PKC-dependent endoplasmic reticulum stress, and ATM/ATR–P53 checkpoints promotes apoptosis and cell-cycle arrest within the neuroepithelium, depleting the progenitor pool required for neural fold elevation and fusion [148,153,154,155,156]. Genetic ablation of key stress mediators, including *Ask1, Jnk1/2*, *Prkcd*, and *Casp8*, significantly reduces NTD incidence in diabetic models, demonstrating that oxidative/nitrosative signaling is not merely associative but mechanistically required for diabetic teratogenesis [153,154,155]. Mitochondrial dysfunction further amplifies this vulnerability, as diabetic embryos exhibit collapse of mitochondrial membrane potential, excessive matrix ROS, and enhanced ER–mitochondrial crosstalk, defects that are rescued by SOD2 overexpression [157,158].

Antioxidants are well known to reduce the severity of oxidative stress and prevent diabetes-induced NTDs. Treatment of in vitro cultured embryos from Cohen diabetic sensitive rats (that develop diabetes under high sucrose low copper diet) with the antioxidants vitamins C and E reduced fetal oxidative stress and prevented neural tube defects [95]. Similar findings were reported by Zaken et al. [159] in rat embryos of the outbred Sabra strain cultured in diabetic culture medium. The addition of nitroxide radicals (that are potent antioxidants) to the diabetic culture medium prevented the NTDs and normalized the redox status of the embryos [96]. Interestingly, Vitamins C and E given in pregnancy to streptozotocin-induced diabetic rats induced a dose-dependent reduction in fetal malformations and oxidative stress [97]. However, these antioxidants did not prevent fetal growth retardation [160].

In parallel with direct cellular injury, diabetic redox stress disrupts epigenetic regulation essential for neurulation. Hyperglycemia alters the activity of DNA methyltransferases, histone-modifying enzymes, and redox-sensitive chromatin regulators, leading to persistent epigenetic “metabolic memory” even after glucose normalization [161,162]. These changes include SET7-mediated H3K4 methylation, loss of repressive H3K9me2/3 marks, and sustained histone acetylation at inflammatory and stress-responsive loci, all of which increase transcriptional noise during a developmental window that requires exceptional epigenetic precision. Such chromatin remodeling is energetically costly and places additional demand on methyl-donor metabolism.

Diabetic embryopathy appears to validate the proposed SAMe-centered convergence model from an endogenous metabolic perspective. Hyperglycemia-induced oxidative and nitrosative stress simultaneously damages neuroepithelial cells, increases antioxidant demand, and restricts one-carbon flux, resulting in SAMe depletion and collapse of epigenetic regulation required for neural tube closure. Together with VPA teratogenicity, these findings demonstrate that distinct chemical and metabolic insults converge on a shared SAMe-dependency, providing a unifying explanation for NTD pathogenesis across diverse environmental and physiological contexts.

## 10. Convergence of Epigenetics and Oxidative Stress During Neurulation: Implications for SAMe Role

The genes essential for dorsoventral patterning and hinge-point formation, including *Pax3*, *Pax7*, *Shh*, *Sox2*, and multiple *Bmp* family members, require precisely regulated DNA methylation, histone methylation/acetylation, and chromatin accessibility to execute their spatiotemporal programs during neurulation [163,164,165]. This epigenetic precision is highly vulnerable to oxidative and nitrosative stress arising during early embryogenesis from mitochondrial respiration, hypoxia-reperfusion episodes, maternal diabetes, inflammation, or teratogen exposure. Elevated ROS and RNS can directly alter the activity of DNMTs, TET dioxygenases, HDACs, and histone methyltransferases/demethylases, thereby changing their catalytic properties and substrate affinity [78,79,80]. Oxidative inhibition of TET enzymes impairs conversion of 5-methylcytosine to oxidized derivatives, promoting locus-specific DNA hypermethylation at regulatory elements of key neurulation genes. At the same time, redox-sensitive cysteine residues in histone-modifying enzymes are susceptible to reversible oxidation or S-nitrosylation, reshaping acetylation and methylation states across neural plate chromatin [78,166]. These epigenetic distortions compromise transcriptional programs governing neural fold dynamics, including proliferation, apical constriction, and midline fusion, thereby predisposing embryos to NTDs [40,57,167].

Although oxidative stress, epigenetic dysregulation, and folate-dependent one-carbon metabolism are each implicated in NTDs, their mechanistic convergence during neurulation has remained incompletely defined. We propose that SAMe depletion represents the key metabolic bottleneck linking redox imbalance to impaired methylation and developmental signaling (Table 3). Under physiological conditions, SAMe supports epigenetic methylation while also allosterically activating cystathionine β-synthase (CBS), thereby promoting transsulfuration flux, cysteine generation, and glutathione synthesis, the embryo’s dominant redox buffer [168,169,170,171]. When oxidative stress increases glutathione demand, homocysteine is diverted away from remethylation, reducing methionine regeneration, collapsing the SAMe:SAH ratio, and lowering methylation capacity at loci such as *Shh*, *Pax3*, and *Pax7* [172]. Prolonged redox stress further depletes SAMe and glutathione, thereby reinforcing both epigenetic instability and antioxidant failure.

Multiple teratogenic and metabolic models support this convergence. VPA reduces methionine and glutathione while elevating homocysteine, creating a strongly oxidized embryonic environment [137,178]. Clinical observations parallel these findings, as mothers with NTDs-affected pregnancies show increased disulfides and reduced thiols, consistent with systemic oxidative pressure [179]. VPA also disrupts mitochondrial function and increases nitrative protein modifications, including NOS2 induction, excessive NO production, protein nitration, and S-nitrosylation, all of which are strongly linked to apoptosis and neural plate destabilization [93,149]. Although NO has protective actions at physiological levels, its excess synergizes with ROS to promote neuroepithelial injury and NTDs [94,180,181]. Similar oxidative and epigenetic signatures are observed in all-trans retinoic acid teratogenesis and diabetic pregnancy, both of which also show methylation defects such as DNMT3B-mediated repression of *Pax3* [108,182].

The centrality of SAMe deficiency is further reinforced by direct experimental studies: inhibition of SAMe synthesis in early embryos collapses the SAMe:SAH ratio, suppresses methyltransferase activity, and causes neural tube closure defects [40]. Ethionine-treated embryos with SAMe depletion exhibit excessive ROS generation, impaired mitophagy through PINK1–parkin disruption, and CASP3 activation, demonstrating that SAMe deficiency amplifies oxidative stress while destabilizing epigenetic control [173]. Low SAMe reduced H3K27me3 by suppressing EZH2 and activating UTX, thereby disrupting β-catenin signaling and neural progenitor differentiation [174]. Homocysteine-induced H3K79 homocysteinylation also disrupts expression of critical neurulation genes such as *Smarca4*, *Cecr2*, and *Dnmt3b* [109]. Likewise, methionine deprivation or methotrexate exposure lowers SAMe availability and impairs PRMT1-dependent arginine methylation, collapsing Wnt signaling required for neural fold elevation [175].

At the biochemical level, transsulfuration depends on SAMe not only as an allosteric activator but also as a stabilizer of CBS protein structure [176,177]. When SAMe declines, CBS stability falls, cysteine availability decreases, and glutathione synthesis weakens. This establishes a self-reinforcing redox–epigenetic failure loop in which rising ROS further impairs methionine regeneration and MAT activity, accelerating SAMe depletion [183,184]. Chronic SAMe deficiency, as observed in MAT1A-deficient models, is sufficient to induce spontaneous oxidative stress, lipid peroxidation, and epigenetic instability [185]. Under inflammatory conditions, reduced MAT activity and GNMT suppression further limit SAMe availability, whereas exogenous SAMe restores glutathione synthesis by re-inducing GCL expression [186]. These findings are consistent with recent evidence that SAMe can mitigate VPA-associated oxidative and developmental injury during neurulation [8].

In diabetic embryos, reduced SAMe availability is accompanied by downregulation of DNMT1, DNMT3A, and DNMT3B, leading to global hypomethylation and repression of insulin-like growth factor signaling pathways critical for embryonic growth and survival [98]. Vitamin B12 supplementation restores SAMe levels, normalizes DNMT expression, and rescues methylation patterns, demonstrating that one-carbon impairment is a causal, reversible contributor to diabetic NTDs. In type 2 diabetes, metformin-associated vitamin B12 deficiency, exacerbated by altered gut microbiota, drives methyl-donor availability, elevates homocysteine, and disrupts folate metabolism, reinforcing SAMe insufficiency as a common biochemical vulnerability [99]. As with VPA exposure, diabetic embryopathy couples redox stress with one-carbon metabolic failure, converging on SAMe depletion as a critical determinant of epigenetic instability.

To test the SAMe-centered dynamic interaction of redox stress and epigenetics, we treated pregnant ICR mice with SAMe on GD 8.5 and 9.5, followed by VPA 1 h later. We examined fetuses on embryonic day 15.5. VPA induced fetal growth retardation, 32.5% exencephaly, and a high resorption rate, and in the brain increased malondialdehyde (MDA) concentrations, increased antioxidant enzyme activity, and elevated expression of antioxidant genes, with increased expression of *NOS1* and *NOS2* genes, implying increased nitrosative stress. SAMe potentially prevented more than 96% of VPA-induced exencephaly, markedly reduced fetal mortality and growth retardation, maintained cellular balance, and prevented peroxidation [8]. Mechanistically, redox pressure increases glutathione demand and diverts homocysteine toward transsulfuration, reduces methionine regeneration, depletes SAMe, elevates SAH, and constrains methyltransferase activity [168,187,188,189,190,191,192].

So far, we have attempted to demonstrate links between oxidative/nitrosative stress with NTD-associated epigenetic abnormalities [108,109,137,178,179,182]. The proposed connection between these observations is that increased GSH demand may favor transsulfuration over remethylation, thereby limiting methionine regeneration and SAMe-dependent methylation, while reduced SAMe may weaken CBS-supported GSH production [168,177]. This reciprocal relationship offers a plausible metabolic explanation for the convergence of redox imbalance, reduced methylation potential, and dysregulated neurulation programs. However, whether redox stress consistently precedes SAMe depletion and whether SAMe restoration prevents the downstream epigenetic and morphogenetic defects remain open experimental questions.

Seminal studies describe physiologically relevant conditions in human pregnancy, where the proposed model potentially operates. In mothers of NTD-affected pregnancies, plasma SAMe is significantly lower and SAH higher, yielding a reduced SAMe:SAH ratio that reflects impaired methyl-donor capacity, alongside elevated homocysteine and oxidized glutathione (GSSG) as markers of oxidative stress [193,194]. These biochemical signatures (low SAMe, elevated SAH, reduced SAMe:SAH ratio, and increased oxidative stress) match the mechanistic expectation that redox pressure diverts one-carbon flux toward transsulfuration, depleting SAMe and elevating SAH, thereby constraining methyltransferase activity at critical neurulation loci [66,193,194].

Evidence from human fetal tissues shows that global DNA hypomethylation and locus-specific methylation defects in NTD brains co-occur with altered maternal one-carbon status. In terminated NTD fetuses, global 5-mC and LINE-1 hypomethylation in neural tissue are associated with lower maternal vitamin B12 and altered folate status, implicating the broader methionine/SAMe axis [48,49,67]. Studies of fetal brain further demonstrate increased histone acetylation (e.g., H4K5ac), altered 5-hmC patterns linked to ROS exposure, and promoter hypermethylation of neurulation genes such as FZD3, consistent with redox-sensitive epigenetic remodeling under limited methyl-donor supply [59,66,88]. The magnitude of SAMe and SAMe:SAH changes reported in human NTD cohorts (~5–10% reduction in plasma SAMe and larger proportional decreases in SAMe:SAH ratio) falls within ranges that in vitro reduce DNMT and histone methyltransferase activity, increase SAH-mediated inhibition, and sensitize cells to oxidative damage [193,194]. Thus, the direction and scale of one-carbon and redox disturbances in human NTDs are consistent with a SAMe-limited, redox-stressed epigenetic environment during neurulation, supporting the view that modest, physiologically attainable reductions in SAMe availability can tip the epigenetic balance at critical neurulation genes and contribute to the multifactorial etiology of human NTDs.

**Figure 4:**
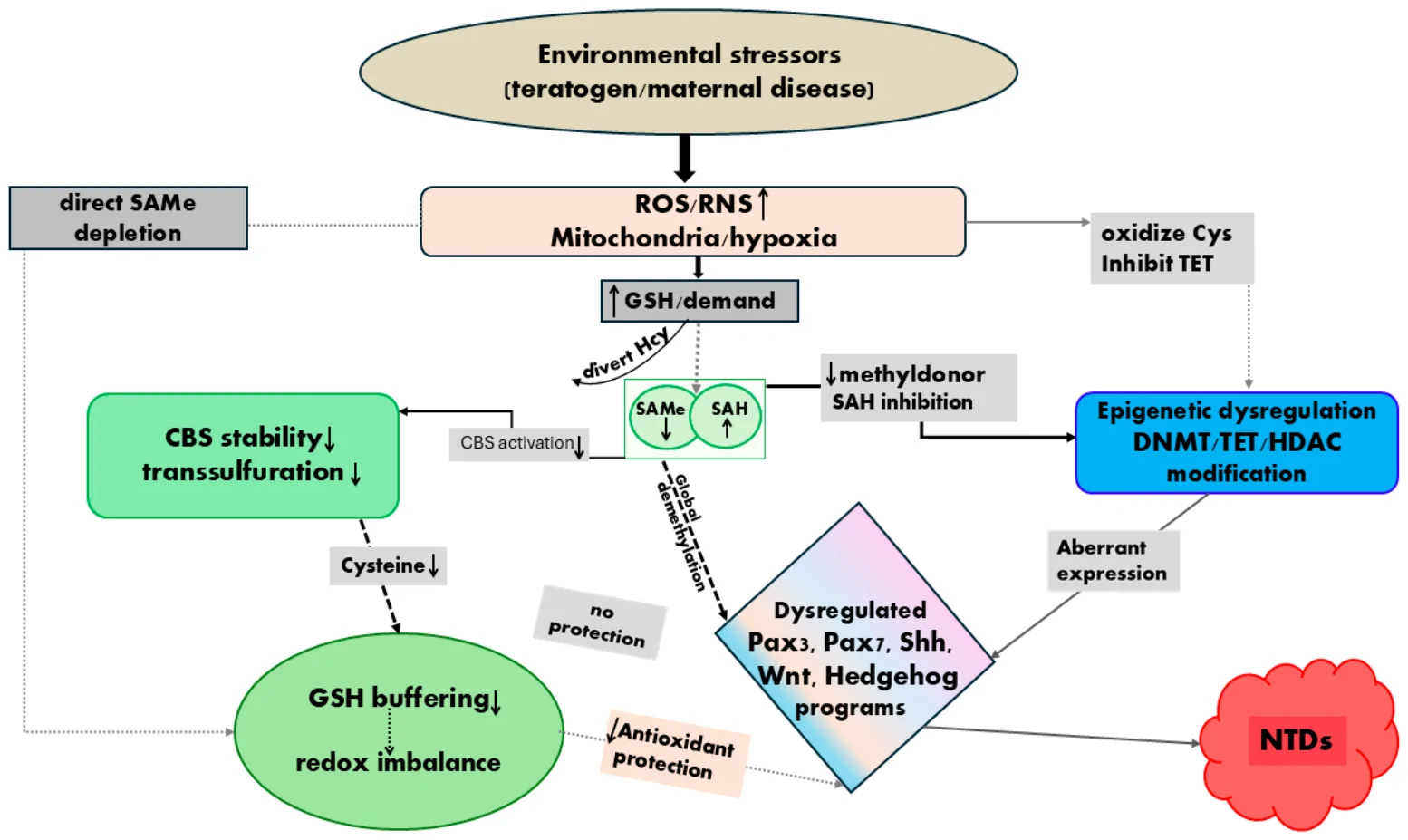
Proposed convergence of redox stress and epigenetic dysregulation on SAMe depletion during neurulation. Maternal or teratogenic stressors, including VPA exposure, diabetes, and inflammation, increase ROS/RNS production and may increase glutathione demand. ROS/RNS can directly modify redox-sensitive epigenetic enzymes, including DNMTs, TET enzymes, and HDACs, while increased antioxidant demand may divert homocysteine toward transsulfuration and away from remethylation. This may reduce methionine regeneration and SAMe availability, increase the relative inhibitory influence of SAH on methyltransferases, and weaken CBS-dependent transsulfuration and GSH production. The resulting redox and epigenetic disturbances may converge on neurulation-associated programs involving Pax3, Pax7, Shh, and Wnt/β-catenin–hedgehog signaling, thereby impairing neural fold elevation and closure and increasing susceptibility to NTDs. Orange nodes indicate stress inputs; green, redox metabolism; blue, epigenetic regulation; red, developmental outcomes. Solid arrows indicate relationships supported by direct biochemical or experimental evidence, whereas dashed arrows indicate proposed or indirectly supported relationships. Arrowheads indicate the direction of the biological effect; inhibitory effects are shown with blunt-ended lines or labelled “inhibits.” The model represents a testable mechanistic hypothesis and does not imply that every connection has been established in a single experimental or human model. Abbreviations: SAMe, S-adenosylmethionine; SAH, S-adenosylhomocysteine; CBS, cystathionine β-synthase; GSH, glutathione; Hcy, homocysteine; NTDs, neural tube defects. Dashed arrows demonstrate relationships for hypothesized interactions.

In summary, Figure 4: oxidative and nitrosative stress during neurulation diverts one-carbon flux toward transsulfuration, lowers SAMe availability, elevates SAH, and oxidatively modifies methylation enzymes. This combination supposedly collapses methylation-dependent gene regulation, weakens glutathione-based antioxidant defenses, and disrupts Wnt/β-catenin, sonic hedgehog and pax3 signaling to destabilize neuroepithelial proliferation and survival. Direct evidence that SAMe deficiency can impair neural tube development through altered histone methylation and β-catenin signaling further strengthens this framework. By positioning SAMe depletion as the rate-limiting nexus linking redox imbalance to epigenetic and morphogenetic failure, this model provides a plausible unifying explanation for how diverse environmental, metabolic, and genetic insults converge to produce neural tube defects [68,69,127].

## 11. Conclusions

Neural tube defects seem to arise from failure of the tightly coordinated transcriptional and morphogenetic programs during neurulation, yet the mechanistic link between diverse genetic, environmental, and metabolic risk factors have remained unresolved. Here, we propose a unifying model in which oxidative and nitrosative stress converge on SAMe depletion as a rate-limiting metabolic bottleneck that translates redox imbalance into epigenetic failure. Although SAMe is not an established therapeutic intervention for prevention of human NTDs, this model positions SAMe as a promising mechanistic and experimental target for translational NTDs’ research and application. During neurulation, DNA and histone methylation require sustained SAMe availability to regulate developmental pathways such as Pax3, Wnt/β-catenin, and hedgehog signaling. However, increased antioxidant demand under redox stress diverts one-carbon flux toward glutathione synthesis, lowering methionine regeneration, elevating SAH, and collapsing the SAMe:SAH ratio, thereby constraining methyltransferase activity and destabilizing chromatin regulation. This mechanism provides a coherent explanation for why distinct insults (including VPA exposure and maternal diabetes) produce overlapping neurulation defects by converging on a shared redox–one-carbon–epigenetic axis. In this framework, SAMe is not merely a methyl donor but a central metabolic integrator of redox buffering, epigenetic fidelity, and developmental signaling, and its depletion may represent the point at which adaptive stress-responses transition into irreversible morphogenetic failure. Definitive validation of this model would require demonstrating that redox stress induces an early decline in embryonic SAMe and SAMe:SAH prior to neural tube closure defects; that this reduction corresponds to locus-specific epigenetic disruption at neurulation genes; and that restoration of SAMe rescues both methylation integrity and neural tube closure in vivo. While these animal and theoretical models provide powerful mechanistic insights, their direct relevance and exact therapeutic potential in human pregnancy require careful translational verification, as observational correlations do not inherently confirm full causation. More could be achieved with integrated quantification of SAMe/SAH/homocysteine/GSH and locus-specific methylation profiling to bridge the gap between experimental findings and clinical reality.

## Figures and Tables

**Table 3:** Supporting models for redox stress–epigenetic intersection in neural tube defects.

Authors	Model/Sample	Main Finding
[88]	Human and mouse neural cultured fetal tissues	Increased ROS was associated with loss of 5hmC in NTD tissue, linking PAH exposure to epigenetic disruption.
[89]	atRA-induced rat NTDs model	Malformed spinal cords showed reduced SOD and GSH-Px, elevated MDA, and increased apoptosis.
[58]	Chick embryo; TSA exposure	HDAC inhibition increased H3/H4 acetylation, induced apoptosis, and caused posterior neuropore closure defects.
[59]	Human NTDs brain tissue	NTDs brains showed elevated histone acetylation, including increased H4K5ac.
[65]	RA-induced mouse model; human anencephaly	Altered H3K27me3 enrichment and dysregulated HOX expression were observed in both animal and human NTDs samples.
[161,162]	Hyperglycemic/diabetic models	Hyperglycemia induced persistent redox-dependent epigenetic remodeling and metabolic memory.
[98]	Diabetic embryopathy model	Reduced SAMe availability was associated with DNMT downregulation, global hypomethylation, and impaired embryonic growth signaling.
[99]	Type 2 diabetes/B12-related metabolic dysfunction	Vitamin B12 deficiency and altered one-carbon metabolism limited methyl-donor availability and increased homocysteine.
[109]	Embryonic mouse model	H3K79 homocysteinylation disrupted expression of neurulation genes including *Smarca4*, *Cecr2*, and *Dnmt3b*.
[173]	Ethionine-induced SAMe depletion model	SAMe depletion increased ROS, impaired mitophagy, and activated caspase 3.
[174]	SAMe-deficient embryonic model	Low SAMe reduced H3K27me3 via EZH2 suppression and UTX activation, impairing β-catenin signaling.
[175]	Methionine deprivation/methotrexate model	Reduced SAMe impaired PRMT1-dependent arginine methylation and collapsed Wnt signaling.
[8]	Mouse model of VPA exposure and SAMe rescue	SAMe pretreatment rescued >96% of VPA-induced exencephaly and restored redox balance.
[93,138]	VPA teratogenesis model	VPA induced NOS2, protein nitration, and S-nitrosylation, promoting apoptosis and neurulation defects.
[176,177]	Biochemical studies of CBS regulation	SAMe stabilizes CBS and promotes transsulfuration flux and GSH production.

Studies are listed in order of appearance in the manuscript and summarize experimental and clinical models that link oxidative/nitrosative stress, one-carbon metabolism, and epigenetic dysregulation to neural tube defects (NTDs). Abbreviations: atRA, all-trans retinoic acid; B12, vitamin B12 (cobalamin); CBS, cystathionine β-synthase; DNMT, DNA methyltransferase; EZH2, enhancer of zeste homolog 2; GSH, glutathione; H3/H4, histone 3/4; H3K27me3, trimethylation of lysine 27 on histone H3; H3K79, lysine 79 on histone H3; H4K5ac, acetylation of lysine 5 on histone H4; HDAC, histone deacetylase; HOX, homeobox genes; MDA, malondialdehyde; NOS2, inducible nitric oxide synthase; NTDs, neural tube defects; PAH, polycyclic aromatic hydrocarbon; PRMT1, protein arginine methyltransferase 1; ROS, reactive oxygen species; SAMe, S-adenosyl-L-methionine; Smarca4, SWI/SNF related, matrix associated, actin dependent regulator of chromatin, subfamily a, member 4; SOD, superoxide dismutase; TSA, trichostatin A (HDAC inhibitor); UTX, ubiquitously transcribed tetratricopeptide repeat X-linked (KDM6A histone demethylase); VPA, valproic acid; Wnt, wingless-related integration site; β-catenin, beta-catenin; 5hmC, 5-hydroxymethylcytosine. Tables have authors arranged as they appear in the manuscript.

## Data Availability

No new data were created or analyzed in this study. Data sharing is not applicable to this article.

## List of Abbreviations

5hmC	5-Hydroxymethylcytosine
5mC	5-Methylcytosine
AMT	Aminomethyltransferase
AP	Anteroposterior
ASD	Autism spectrum disorder
atRA	All-trans retinoic acid
ATP	Adenosine triphosphate
BaP	Benzo[a]pyrene
BMP	Bone morphogenetic protein
CAT	Catalase
CBS	Cystathionine β-synthase
CBP	CREB-binding protein
cGMP	Cyclic guanosine monophosphate
Cdk2	Cyclin-dependent kinase 2
Cdk4	Cyclin-dependent kinase 4
Cecr2	Ccctc-binding factor-like protein 2
CpG	Cytosine–phosphate–guanine
DMR	Differentially methylated region
DNMT	DNA methyltransferase
DNMT1	DNA methyltransferase 1
DNMT3A	DNA methyltransferase 3A
DNMT3B	DNA methyltransferase 3B
EMT	Epithelial–mesenchymal transition
FGF	Fibroblast growth factor
FZD3	Frizzled class receptor 3
GD	Gestational day
GLDC	Glycine dehydrogenase
GSH	Glutathione
GSH-Px/GPx	Glutathione peroxidase
HAT	Histone acetyltransferase
Hcy	Homocysteine
HDAC	Histone deacetylase
HDI	Histone deacetylase inhibitor
H3K4ac	Histone H3 lysine 4 acetylation
H3K9ac	Histone H3 lysine 9 acetylation
H3K18ac	Histone H3 lysine 18 acetylation
H3K27ac	Histone H3 lysine 27 acetylation
H3K27me3	Histone H3 lysine 27 trimethylation
H3K79	Histone H3 lysine 79
H4K5ac	Histone H4 lysine 5 acetylation
IGF2	Insulin-like growth factor 2
iNOS	Inducible nitric oxide synthase
LINE-1	Long interspersed nuclear element 1
MAT	Methionine adenosyltransferase
MAT1A	Methionine adenosyltransferase 1A
MDA	Malondialdehyde
METTL3	Methyltransferase-like 3
MTHFD1L	Methylenetetrahydrofolate dehydrogenase 1-like
MTHFD2	Methylenetetrahydrofolate dehydrogenase 2
MTHFR	Methylenetetrahydrofolate reductase
N-Cadherin	Neural cadherin
NADPH	Nicotinamide adenine dinucleotide phosphate
NDDs	Neurodevelopmental disorders
NO	Nitric oxide
NOX	NADPH oxidase
Nrf2/NRF2	Nuclear factor erythroid 2–related factor 2
NSCs	Neural stem cells
NTDs	Neural tube defects
O_2_^−^	Superoxide anion
–OH	Hydroxyl radical
ONOO^−^	Peroxynitrite
PAHs	Polycyclic aromatic hydrocarbons
PCNA	Proliferating cell nuclear antigen
PCP	Planar cell polarity
PINK1	PTEN-induced kinase 1
PRMT1	Protein arginine methyltransferase 1
RA	Retinoic acid
RNS	Reactive nitrogen species
ROS	Reactive oxygen species
SAH	S-Adenosylhomocysteine
SAMe	S-Adenosylmethionine
SHH/Shh	Sonic hedgehog
SHMT2	Serine hydroxymethyltransferase 2
SOD	Superoxide dismutase
SOD2	Superoxide dismutase 2
TET	Ten-eleven translocation
TNF	Tumor necrosis factor
TSA	Trichostatin A
UTX	Ubiquitously transcribed tetratricopeptide repeat protein, X chromosome
VPA	Valproic acid
Wnt	Wingless-related integration site
